# The Effects of Mixed *Fusarium* Mycotoxins at EU-Permitted Feed Levels on Weaned Piglets’ Tissue Lipids

**DOI:** 10.3390/toxins13070444

**Published:** 2021-06-27

**Authors:** Omeralfaroug Ali, Miklós Mézes, Krisztián Balogh, Melinda Kovács, András Szabó

**Affiliations:** 1 Department of Physiology and Animal Health, Institute of Physiology and Nutrition, Hungarian University of Agriculture and Life Sciences, Kaposvár Campus, Guba S. u. 40., 7400 Kaposvár, Hungary; kovacs.melinda@uni-mate.hu (M.K.); szan1125@freemail.hu (A.S.); 2 Department of Feed Toxicology, Institute of Physiology and Nutrition, Hungarian University of Agriculture and Life Sciences, Gödöllő Campus, Páter K. u. 1., 2053 Gödöllő, Hungary; mezes.miklos@uni-mate.hu (M.M.); balogh.krisztian.milan@uni-mate.hu (K.B.); 3 MTA-KE-SZIE Mycotoxins in the Food Chain Research Group, Department of Physiology and Animal Health, Institute of Physiology and Nutrition, Hungarian University of Agriculture and Life Sciences, Kaposvár Campus, Guba S. u. 40., 7400 Kaposvár, Hungary

**Keywords:** fusarium, deoxynivalenol, zearalenone, fumonisin B series, pigs, phospholipids, oxidative stress, clinical chemistry, co-exposure

## Abstract

At exactly the individual permitted EU-tolerance dietary limits, fumonisins (FB: 5 mg/kg diet) and mixed fusariotoxins (DZ: 0.9 mg deoxynivalenol + 0.1 mg zearalenone/kg diet, and FDZ: 5 mg fumonisins + 0.9 mg deoxynivalenol + 0.1 mg zearalenone/kg diet) were administered to piglets (*n* = 6/group) for three weeks. Bodyweights of intoxicated piglets increased, while feed conversion ratios decreased. In FDZ, both the absolute and relative weight of the liver decreased. In the renal-cellular membrane, the most pronounced alterations were in FDZ treatment, followed by individual FB exposure. In both treatments, high proportions of C20:0 and C22:0 with low fatty acid (FA) unsaturation were found. In hepatocyte phospholipids, FDZ toxins exerted antagonistic interactions, and FB had the strongest increasing effect on FA monounsaturation. Among all investigated organs, the spleen lipids were the least responsive, in which FDZ expressed synergistic reactions on C20:0 (↑ FDZ vs. FB) and C22:0 (↓ FDZ vs. DZ). The antioxidant defense of the kidney was depleted (↓ glutathione concentration by FB-exposure). Blood plasma indicated renal injury (profound increase of urea and creatinine in FB vs. DZ and FDZ). FB strongly increased total-cholesterol and low density lipoprotein concentrations, whereas FDZ synergistically increased gamma-glutamyltransferase, alkaline-phosphatase, calcium and phosphorus levels. Summarized, individual and combined multiple fusariotoxins modified the membrane lipid profile and antioxidant defense of splanchnic organs, and serum biochemicals, without retarding growth in piglets.

## 1. Introduction

Mycotoxins are the toxic secondary metabolites of fungi and toxigenic molds, and they represent global health and economic challenges that are estimated to increase due to global climate change [1]. To date, more than 400 toxic metabolites have been isolated and structurally characterized. Among them, fusariotoxins (deoxynivalenol (DON), fumonisins (FBs) and zearalenone (ZEN)) are the most prevalent toxins in feed commodities [2,3]. Fusariotoxins are mainly produced by the large genus *Fusarium* (known for more than 100 years), although some other species can also produce some of its toxins. Their toxicity mechanism in animals is complex and varies according to species, organ and toxin type. Based on the fusariotoxins’ mode of action, health concerns have been investigated in swine and other species.

Deoxynivalenol (DON or vomitoxin) is the most abundant trichothecene type toxin found in grain, and its mode of action relies on the interaction with the active site of peptidyl-transferase on ribosomes, thus inhibiting protein and nucleic acid synthesis, triggering the ribotoxic stress response and altering cellular signalling [4]. In swine, DON exposure (1–3 mg/kg diet) affects the gastrointestinal tract (GIT) and causes diarrhoea, nausea, reduced feed intake and weight gain [4,5]. Zearalenone (ZEN) is a resorcylic acid lactone mycotoxin that has structural analogy oestrogens. ZEN exerts oestrogenic activity (high affinity to the cytosolic 17β-oestradiol receptors; ERα and ERβ) that is responsible for its toxic mechanism [6], although it is possibly not associated with oestrogenic activity [7,8]. Therefore, as well as its ability to affect the reproductive system (ZEN > 1 mg/kg diet), it also affects non-reproductive systems, exerting hepatotoxic, haemotoxic, immunotoxic and genotoxic effects [9]. Fumonisins (FUM) are comprised of the A, B, C and P series, whereas fumonisin B (FB, which includes FB_1_, FB_2_, FB_3_, FB_4_, FB_5_ and FB_6_) is the most widespread and studied series, especially the FB_1_ analogue. FB has structural similarities to sphingoid bases (namely sphinganine (Sa) and sphingosine (So)) so it disrupts sphingolipid metabolism by altering the ratio between Sa to So and inhibiting ceramide synthase (CerS), a rate-limiting enzyme for ceramide biosynthesis that is a precursor for complex sphingolipids [10]. In pigs, the heart, lung, liver and kidney are the primary target organs for FB > 3.7 mg/kg (Terciolo et al., 2019), whereas under higher doses, pulmonary oedema and cardiac dysfunction are frequent clinical symptoms [11].

The adverse effects of fusariotoxin were the key factors behind the establishment of guidance limits (lowest observed adverse effect level) in food and feed (e.g., 2006/576/EC [12]). The maximum allowed levels of DON, ZEN and FB in a complete diet intended for piglet feeding are 0.9, 0.1 and 5 mg/diet, respectively [12]. According to a recent report [2], DON, ZEN and FB are highly co-occurring in mixtures, whereas DON and ZEN levels are correlated in maize and wheat. Despite the risk assessment approach for mycotoxin mixtures in food and feed that has been recently proposed by EFSA [13], their interaction is not yet clearly understood. The co-exposure of animals to several mycotoxins is highly important, ultimately contributing to the prediction process (i.e., additive, synergistic, or antagonistic). The interactive effects of the tertiary fusariotoxins mixture have not been intensively studied, especially in vivo, and only scarce reports are available [14,15,16,17,18,19,20,21,22]. Among toxicological assessment parameters, FA metabolism is receiving profound interest, since lipids are associated with numerous diseases and can provide hints about cells’ physiological status [23,24]. The majority of in vivo and in vitro studies investigated the effects of individual FB_1_/FB on the hepatic membrane lipids of rats [23,25,26,27,28,29,30,31,32] and, to a lesser extent, on the blood of rabbits and the blood and liver of piglets [33,34]. Only a single study investigated the interaction of fusariotoxin (DON, ZEN and FB_1_; individual, and binary and tertiary mixtures) with bilayer lipids of the liver and kidneys of male Wistar rats [15]. Swine have not yet been investigated intensively; they are sensitive to fusariotoxins and represent a suitable biomedical model for human toxicology. Therefore, swine represent a good model for our study design.

The primary aim of our study was to investigate the interactive effects of fusariotoxins (namely DON, ZEN and FB), at exactly the individual permitted levels of the 2006/576/EC [12], on the total phospholipid fatty acid (FA) profile of the liver, kidneys and spleen of weaned piglets and, furthermore, to examine whether or not lipid modifications are related to oxidative stress. We also examined the biochemical molecules of the blood plasma, thus assessing the possible presence of hepatotoxicity and nephrotoxicity.

## 2. Results

### 2.1. Growth Performance, Absolute and Relative Organ Weights

The absolute bodyweight (BW) increased with age in the control, while the weekly average bodyweight gain (AWG) was decreased (Table 1). Mycotoxin contaminated diets (FB, DZ and FDZ) improved the AWG (increasing AWG values during the study period) of piglets. The FDZ treatment reduced the absolute and relative liver weights. None of the other measured organ weights (heart, kidney, lung and spleen) were altered.

As shown in Table 2, in feed intake, no significant difference was proven between the different treatments (neither weekly nor for the entire period). The FCR overall values of fusariotoxin exposed groups were decreased (i.e., lower values were attained). This decrease in overall FCR resulted from the low FCR values at the third week, which was statistically proven.

When FB and DZ interactions were assessed, their effects on the BW, BWG and feed conversion ratio (FCR) were antagonistic statistically. Regarding the alteration in liver weight, the FDZ interaction was synergistic on the absolute liver weight, whereas antagonistic on the relative weight.

### 2.2. Kidney Total Phospholipid Fatty Acid Profile

The results of the FA profile of the renal cellular membrane are presented in Table 3. The FB exposure increased the C14:0 (myristic acid) and C16:0 (palmitic acid) proportions. The palmitic acid proportion was also increased by FDZ exposure. Fusariotoxin-treated groups showed higher C20:0 and C22:0 (arachidic and behenic acids, respectively) proportions than the control. FB and FDZ-exposures increased the total saturation (SFA) level and decreased the unsaturation index (UI). The DZ treatment decreased the C24:0 (lignoceric acid) proportion. Despite the total monounsaturated FA proportion (MUFA) not being responsive to any of the treatments, the C16:1n7 (palmitoleic acid) proportion was increased in the FDZ fed group, whereas the C22:1n9 (erucic acid) proportion decreased in the FB and FDZ groups. Compared with the control, the FDZ exposure decreased the C22:5n3 (docosapentaenoic acid, DPA) proportion, whereas the C22:6n3 (docosahexaenoic acid, DHA) proportion was decreased in the renal phospholipids of piglets exposed to FB. FDZ exposure also decreased C20:2n6 (eicosadienoic acid) and C20:3n3 (eicosatrienoic acid) proportions. FDZ-fed groups had low proportions of total polyunsaturation (PUFA) and total omega-3 FAs, and a high ratio of omega-6/omega-3, as well as in the DZ group. The total proportion of omega-6 FAs increased in the DZ fed group as compared to FDZ. A recorded significant decrease in the average chain length was confirmed in DZ and FDZ treatments as compared to control or FB groups.

Fusariotoxins (FDZ) interacted mostly antagonistically with many FAs in the renal phosphatides, such as on myristic, palmitic, palmitoleic, arachidic, behenic, DHA and lignoceric acids. In contrast, for FDZ, synergistic interactions were present in shaping the eicosadienoic acid and DPA proportions. Additive interaction was proven for FB and DZ toxins with eicosatrienoic acid. The ternary combination (FDZ) interacted antagonistically with total saturation values, unsaturation, omega-3 FAs, omega-6 FAs, average chain length and the omega-6 FAs to omega-3 FAs ratio. Total polyunsaturation was shaped by a synergistic effect of the FB and DZ toxins.

**Figure 1:**
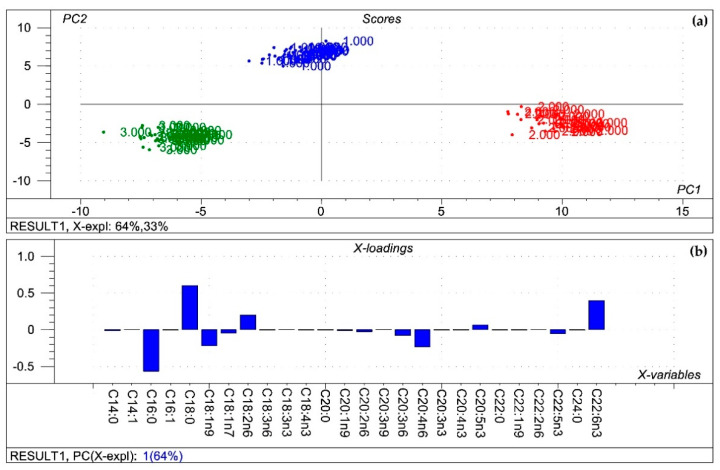
Results of the principal component analysis performed on the compositional data of the membrane fatty acids of organs from all treatments. (**a**) Score plot describes the orientation of the samples belonging to the different organs (1: kidney; 2: liver; 3: spleen) in the plane of the 1st and 2nd principal components (PC1 and PC2, respectively), where PC1 and PC2 are influenced by the multivariate data of the organ phospholipid fatty acids. PC1 and PC2 explain 64% and 33% of the total variance of the membrane fatty acids of the organs, respectively. From the principal component analysis, the polar fatty acid pool of tissues provided a perfect spatial separation of groups, referring to variation in their fatty acid profiles; (**b**) Loading bar graph of the PC1 shows the contribution of the individual liver phospholipid fatty acids to the newly developed latent variable: the higher the loading value, the higher impact of the variance of the respective fatty acid on the variance of PC1. From the loadings, the remarkable fatty acids that contributed to variance between organs are C16:0, C18:0, C18:1n9 and C18:2n6, C20:4n6, C22:6n6.

**Figure 2:**
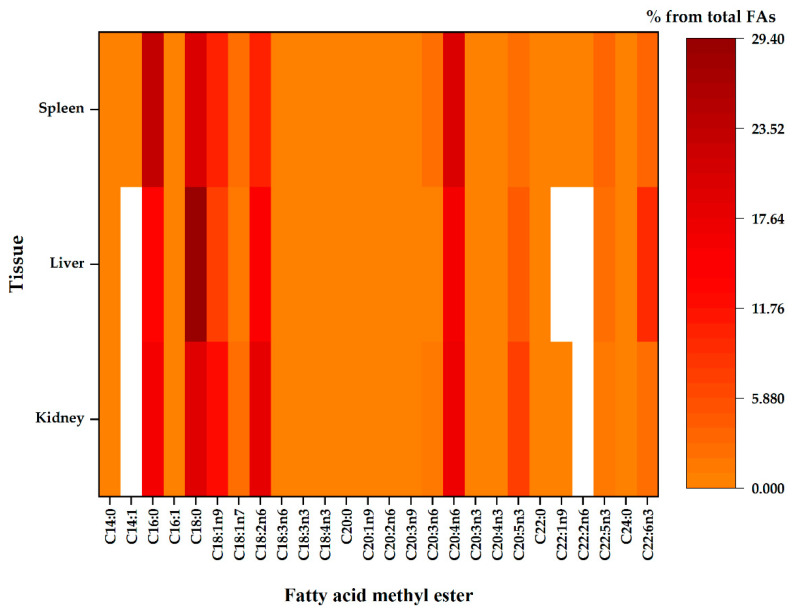
The compositional data of the membrane fatty acids from different tissues, namely the kidney, liver and spleen. Regardless of the tissue, fatty acid proportion increases with colour intensity, whereas the white colour represents fatty acid methyl ester below the detection limit of the GC.

**Figure 3:**
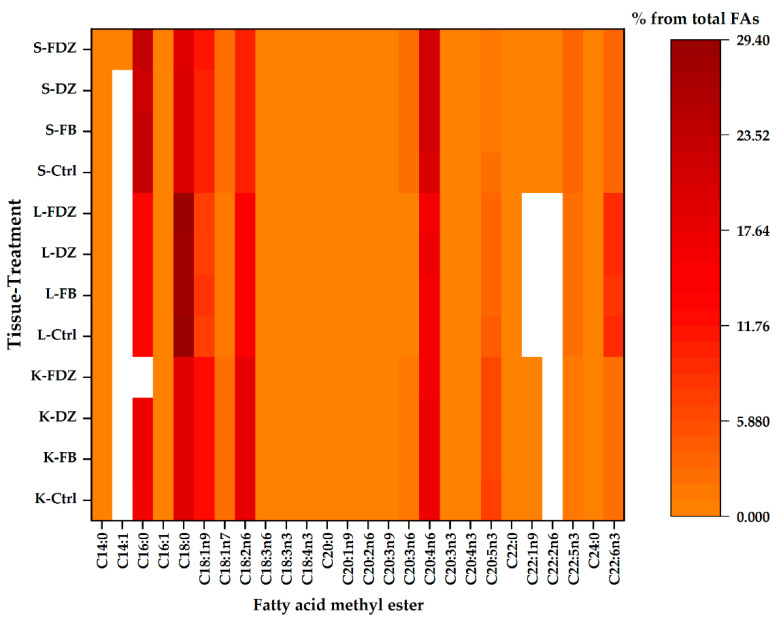
The compositional data of the membrane fatty acids of all treatments from all tissues. The fatty acid proportion increases with colour intensity, whereas the white colour represents fatty acid methyl esters below the detection limit of the GC. (K: kidney; L: liver; S: spleen; Ctrl: control; FB: fumonsins; DZ: deoxynivalenol + zearalenone; FDZ: fumonsins + deoxynivalenol + zearalenone).

Visualisations of the most pronounced FAs responsible for variations between organ FA profiles are presented in Figure 1. Figure 2 and Figure 3 expresses the proportional presence of each FA from the different organs, based on color intensity. 

### 2.3. The Liver Total Phospholipid Fatty Acid Profile

The fatty acid profile of hepatocellular membrane lipids is shown in Table 4. Among all SFAs, myristic acid proportion increased in the FB group. In contrast, DZ and FDZ exposures decreased behenic acid proportion, whereas FB exposure decreased the C18:0 (stearic acid). FB led to a remarkable increase of the monounsaturated FAs (palmitoleic acid and C18:1n9 (oleic acid)). The total omega-3 proportion was shown to decrease, which was associated with the DHA proportional decrease. FB also increased the C18:4n3 (stearidonic acid) proportion when compared to the DZ-fed group. From the calculated indices, total saturation increased in the FDZ-group. In the FB group, total proportions of unsaturation and monounsaturation increased, whereas total proportions of PUFA/MUFA, omega-3 FAs, UI and ACL decreased as compared to other treatments.

Interaction testing revealed antagonistic reactions with FAs and FA indices for all three toxins, providing significantly altered proportions (with ANOVA).

### 2.4. The Spleen Total Phospholipid Fatty Acid Profile

Among all the investigated tissues, the spleen was the least responsive (Table 5) in terms of intoxication. FB exposure was shown to decrease the arachidic acid proportion as compared to FDZ. An intergroup difference between DZ and FDZ groups was found in the lignoceric acid proportion; the lowest proportion was detected in FDZ piglets. The DZ group had a lower behenic acid proportion as compared to FB and FDZ groups. Among all unsaturated FAs, only the stearidonic acid proportion increased (in the FDZ group).

In the FB group, the total proportion of omega-3 FAs decreased and the omega-6/omega-3 ratio increased. A difference in the ACL was present between FB and DZ groups, with DZ providing the higher values.

The fusariotoxins’ results revealed antagonistic reactions for all altered FAs and the calculated FAs indices for all three toxins.

### 2.5. Plasma Nitrogenous Compounds

The piglets’ plasma nitrogenous metabolite data are shown in Table 6. Neither the total protein nor the albumin level was significantly different between fusariotoxin treatments. During the investigated periods, urea and creatinine concentrations were increased by FB, as compared to the control.

With the plasma urea, creatinine and total bilirubin levels, the FB-DZ interaction was antagonistic.

### 2.6. Plasma Lipid Metabolites and Glucose

The results of the plasma lipid metabolites can be seen in Table 7. The plasma total cholesterol concentration was highest in the FB-fed group during the second week, whereas it was lowest in the DZ group. The low density lipoprotein (LDL) concentration was altered during the second week. The highest LDL concentrations were shown in piglets fed on an FB-contaminated diet, while the lowest concentrations were in those fed with DZ. Neither triglycerides (TGs) nor high density lipoprotein (HDL) concentrations responded to fusariotoxin(s) exposures. Glucose remarkably decreased with time, although no intergroup differences were found between treatments.

The FB–DZ antagonistic effect was proven for CHO and LDL.

### 2.7. Plasma Enzyme Activities

Results of the plasma enzyme activities are presented in Table 8. Alterations were detected in the gamma–glutamyl transferase (GGT) and alkaline phosphatase (ALP) activities. After three weeks, the FDZ diet increased ALP activity as compared to the DZ diet. GGT activities began to change after the second week, whereas FB and FDZ groups had the highest activities during the second and third weeks, respectively.

The effect of fusariotoxins (FDZ) on the plasma GGT activity was synergistic after the second week and antagonistic in the third week. FDZ exerted a synergistic effect on the serum ALP activity after three weeks.

### 2.8. The Plasma Ion Concentrations

Plasma ion concentrations are shown in Table 9. The calcium (total and corrected (ionized) levels at 21 days of the FDZ fed piglets have shown the highest (total and corrected/ionized) values as compared to the control and DZ groups. Phosphorus (P) concentration decreased after seven days in the DZ and FDZ groups, whereas only the FDZ group maintained its highest level at 21 days.

Interactions between fusariotoxins varied among the different ions in the blood plasma. Synergistic effects were recorded for total calcium (Ca) and corrected calcium (Corr. Ca). In contrast, fusariotoxins exhibited an antagonistic interaction with phosphorus concentration.

### 2.9. Antioxidants and Lipid Peroxidation

Most tissues (plasma, liver and lungs) did not show differences between treatments in terms of antioxidant capacity (Table 10). However, inter-group differences could only be detected in the kidney. Alterations were confirmed in the GPx activity (decreased by FB and FDZ exposure) and MDA concentration (lowered by FB exposure).

In the renal tissue, the fusariotoxins’ interaction was antagonistic with the MDA concentration, whereas it was additive for the GPx activity.

## 3. Discussion

### 3.1. Growth Performance, Absolute and Relative Organ Weights

Fusariotoxins frequently contaminate swine feed and consequently alter animal performance and compromise health. To our best knowledge, the interactive effects of the tertiary mixture of fusariotoxins (FB, DON and ZEN) on piglets have not yet been investigated *in vivo*. In the present study, mortality was not present, possibly due to the relevant fusariotoxin tolerance limits [12]. Remarkably, fusariotoxin exposures altered the piglets’ growth potential, that is, the BWG and FCR were improved. The administration of FDZ had a synergistic effect on lowering the absolute liver weight. Our finding is dissimilar to other in vivo findings reported in rats [15,16,22] and rabbits [20], where fusariotoxins (individual and combined) did not alter the BWG nor the feed intake. The beneficial effects on growth observed during fusariotoxins’ exposures are mainly observed because of a decrease in AWG over time in the control animals. At some stage of life, the AWG (as expressed on a weekly basis) slows down with aging in all animals [35], which is mostly nonlinear, except in fish [36]. We have associated high BWs with improvements in FCRs, but the real physiological background of this phenomenon remains unknown. A plausible explanation may be some non-systematic oedema caused by fusariotoxin exposure; in swine, the lungs, liver, heart, gut, kidneys, genitals and the immune system are the major targeted tissues for fusariotoxins [5,11,37,38], also reporting oedema.

The outcome of DON + ZEN and FB + DON + ZEN exposures on piglets was unexpected, since at low dietary concentrations, DON could impair feed intake and gastrointestinal functionality [5,39,40]. Piglets probably developed a mechanism that is adaptive to the presence of DON in the diet, or their gastrointestinal microbiome was altered [41] into one with a high de-epoxidation rate. Our findings indicate that, either 0.9 mg DON (under 2006/576/EC guidance) is not relevant for curtailing the piglets’ feed intake under optimal production conditions, or ZEN and/or FB have mimicked the DON-effect (practically, this corroborates the EC guidance level to be reasonable). On the contrary, 0.26 mg ZEN (a fattening pig ZEN-tolerable limit) with 1 mg DON decreased diet intake in piglets [39]. It is worth noting that exposure to low levels of ZEN exerts steroidal properties that stimulate proliferative processes of the digestive tract and may increase bodyweight gain over time, which has been proven in pre-pubertal gilts [42]. A similar finding was attributed to dietary tolerance, or the compensatory effect [43]. We suppose that the inconsistent outcomes may arise from the ratio between toxins, applied doses and age at exposure. Such an establishment requires further analysis, since, up to date, the toxicity mechanism of the combined fusariotoxins is not comprehensible.

### 3.2. The Kidney Total Phospholipid Fatty Acid Profile

To our knowledge, no other in vivo study has investigated the ternary fusariotoxin combinations’ effects on the membrane FA profile of piglet kidneys. Renal tissue from a male Wistar rat shows that individual and/or joint fusariotoxins can alter organ weight, cell membrane lipid profile and redox system stability (glutathione peroxidase). Our study provided findings in piglets that are consistent with those of rats [15,25].

Despite the relative depletion of arachidic and behenic acids by FB and FDZ exposures, the palmitic acid proportions increased and, consequently, the total proportion of SFA increased. SFAs are minimally susceptible to oxidation and, thus, the limitation of FA oxidation restricts mitochondrial oxidative phosphorylation and augments ROS generation, events that contribute to tubular epithelial cell death, oxidative stress and inflammation [44]. High intraperitoneal FB_1_ doses (equivalent to 20, 50 and 100 mg/kg) for five days in male Wistar rats elevated the palmitic acid proportion in renal membrane lipids [25]. Palmitic acid is a precursor for de novo ceramide synthesis through the formation of d18:1 sphinganine. Differences among ceramide species depend on the sphinganine acylation to fatty acyl chains that vary in chain length. FB_1_ is the most toxic analogue and is known to compete with the sphingosine N-acyltransferases and thus disrupt ceramide and sphingomyelin synthesis, in which SFAs are crucial building blocks [45,46]. Thus, proportional depletion of arachidic (half proportional decrease) and behenic acids may indirectly refer to a possible imbalance in the biosynthesis of ceramides and sphingomyelins, a typical FB_1_ mode of action. The novelty of this study is that FB (individual and joint) depleted proportions of arachidic and behenic acids in piglet kidneys have not proven in male rats exposed to pure FB_1_ and FB_1_ joint with DON and ZEN [15], although the rat kidney is the target organ for FB_1_ [47].

Insignificant findings of essential FAs (C18:2n6 (LA) and C18:3n3 (ALA)) among all examined tissues support identical dietary uptake (as also measured directly). Despite tissue LA and ALA being non-responsive to intoxication, their long-chain derivatives were proportionally altered in the kidney by FB and FDZ exposures. Among PUFAs, eicosadienoic, eicosatrienoic acids and DPA decreased in the FDZ group, whereas DHA decreased in the FB group. In the piglet renal tissue, the FB effects on PUFA seemed to be more profound when FB was combined with DON and ZEN. The depletion of omega-3 and omega-6 FAs by FDZ exposure was accompanied by a decrease in their total FA sums, total PUFA and UI, suggestive of the oxidative stress commencement induced by the slight nephrotoxicity [44]. Those FA indices were not responsive to FDZ mixture (following limits of the 2006/576/EC) in the rat kidney [15], but Szabó et al. [25] have presented the dose-dependent decrease in PUFA, omega-3, omega-6 and UI values as illustrated in the rat renal PC after five days of high FB_1_-intraperitoneally exposure (equivalent to more than 20 mg/kg diet). We thus suppose that FB (as a single agent) can modify/perturb the unsaturated acyl chain distribution into the *sn-2* position of polar lipids in piglet renal tissue, whereas a minor effect was achieved when other fusariotoxins were used. This modulation is commonly achieved by disrupting the CerS species that regulate the desaturase enzyme groups [30,31]. 

The total MUFA proportion was non-responsive to any of the treatments, although palmitoleic and erucic acids have been shown to undergo a proportional decrease in the FDZ group. Erucic acid was also decreased by FB exposure. In male Wistar rats exposed to fusariotoxins for 14 days, the erucic acid proportion was increased by ZEN treatment [15]. When rat renal phosphatide sub-classes (i.e., PC, PI and PE) were investigated in a time-series [25], FB_1_ doses above 50 mg/kg diet for five days increased palmitoleic acid and decreased erucic acid proportions, while both FAs increased after ten days of exposure. Thus, we suppose that the MUFA proportional shift relies on the relationship between cellular protective capacity and cytotoxicity degree.

### 3.3. The liver Total Phospholipid Fatty Acid Profile

Similar to in the kidney, the effects of the ternary fusariotoxin (FDZ) exposure on the porcine hepatic membrane lipids have not yet been investigated (neither in vitro nor in vivo), whereas a single study is available on male Wister rats [15]. Alterations in the hepatic membrane lipids induced by FB, especially FB_1_, are well proven in rats (in vitro by [23] and in vivo by [25]) and piglets [34]. The authors associate those modifications with hepatotoxicity. The novel point from the study of Ali et al. [34] is the applied low FB dose, in accordance with the guidance [12]. Remarkably, even though we noticed no hepato-histomorphological alterations (data not shown in this study), the membrane bilayer lipids were modified. We thus propose that alterations in the membrane FAs occur earlier than the histomorphological changes.

FB exposure elevated the myristic acid proportion but depleted the stearic acid proportion, and the total saturation decreased. Neither the accretion of myristic acid proportion nor the depletion of stearic acid proportion was proven in the piglet liver (total phospholipid and ceramide FA profiles) after FB oral exposure, at a high dose ([34], 20 mg/kg diet) or at a low dose ([29], 1.5 mg/kg diet). In the rodent liver, in both in vivo and in vitro studies, the stearic acid response to FB was inconsistent. Most studies showed a proportional increase [15,30,31], while a few studies showed no response [23,32]. Two studies reported the proportional lowering of stearic acid in the rat liver–PC fraction as induced by high FB_1_-doses (in vitro study [26] and in vivo study [28]). Furthermore, due to the high proportions of palmitoleic and oleic acids, total MUFAs were increased in the FB-group. MUFAs are less prone to peroxidation; high MUFAs might exhibit anti-apoptotic and antioxidant (some protective) properties that protect against the oxidative damage of hepatocytes. We thus assume that the high total MUFA ratio compensated for the reduction of total SFA to support the unique membrane rigidity/fluidity and protect against lipid peroxidation. This seems to be manifested by the low PUFA/MUFA and low UI in the FB group.

In the liver, the non-altered essential FAs support our earlier hypothesis of identical feed intake. Among the polyunsaturated omega-3 FAs, stearidonic acid proportion decreased in the DZ group, whereas the DHA proportion decreased in the FB group. A consequence of the DHA proportional depletion was the decrease of total omega-3 FAs in the FB group. In our earlier study, a 20 mg FB/kg diet decreased the DHA proportion in piglet liver, where oxidative stress was the induction mechanism [34]. In this study, the altered FAs were independent of oxidative stress, suggesting an alternative pathway leading to the decrease in the omega-3 FA proportion, besides the already known oxidative route.

### 3.4. The Spleen Total Phospholipid Fatty Acid Profile

The spleen is the largest pool of lymphoid tissue in vertebrates and serves as a blood filter, so it has vital rules for erythrocyte circulation and the immune system [48]. Our spleen data represent secondary data, since the heart, lung, liver and kidney are the primary and most investigated organs. We are not aware of any study investigating the interactive effects of fusariotoxins on the piglet spleen membrane-lipids.

Briefly, when the spleen FA profile was compared to those of the kidneys and the liver, the spleen was found to be the least responsive tissue to fusariotoxins’ exposure. Similar to the liver, FAs’ alterations in the spleen occurred without any lesion in the histological dataset (data not shown). The most abundant FAs in the spleen phospholipids were arachidonic, palmitic, stearic and oleic acids, similar to findings in rat splenocytes’ [49]. Among all SFAs, only the LCSFAs responded to fusariotoxin exposures. The synergistic interaction of FDZ increased the arachidic acid. Furthermore, the proportion of behenic acid was decreased by FB exposure as compared to the DZ. Behenic acid was also synergistically decreased in the FDZ group, as well as the lignoceric acid proportion. However, modifications in LCSFAs did not compromise the total saturation level, but the average FA chain length (ACL) decreased in the FB group. The proportional reduction in LCSFAs refers to the down-regulation of CerS activity in the spleen by FB mycotoxins. Notably, the effects of FB on the spleen were augmented by the addition of DZ, proved by the synergistic effects on the levels of arachidic, behenic and stearidonic acids. The literature suggests that mycotoxins at a tolerable level do not exert toxic effects alone; their toxicity effect is augmented by combinations [50].

Total omega-3 FA levels decreased in the FB group and, consequently, the omega-6 to omega-3 ratio increased. In this study, two scenarios may lead to the lowering of total omega-3 FAs. These are the induction of oxidative stress and the disruption of the desaturase enzymes. The altered omega-6 to omega-3 ratio may indicate the disruption of enzymes that regulate lipid metabolism. Regarding oxidative stress, FB_1_-intravenous injection (1.5 mg/kg BW) in rats induced oxidative stress and DNA breaks in the spleen and liver [51]. None of the proposed events were investigated by us; therefore, the proof of those assumptions requires further investigation. To summarize our findings, modifications of the spleen bilayer lipids induced by fusariotoxins were negligible.

### 3.5. Plasma Nitrogenous Compounds

Plasma nitrogenous compounds’ major protein fractions are albumin and globulins; their unchanged concentrations refer to well-functioning glomerular filtration.

We detected elevated concentrations of urea and creatinine levels in the blood plasma of the FB-fed group, as compared to the control. FB_1_ is well-recognized to cause nephrotoxicity and hepatotoxicity in swine [45]. It was found [45] that even four weeks on a higher dose than this (8.1 mg FB/kg diet) could not alter the urea concentration in piglet plasma, whereas the 12.2 mg FB/diet achieved this. A positive association between plasma urea level and sphingosine-phosphate concentration was also reported—a typical FB mode of action. The novelty of our study is that FB at a dose of 2006/576/EC [12] for three weeks could alter some of the serum nitrogenous compounds. Creatinine is generally handled as a muscular mass biomarker. However, renal efficiency has a remarkable impact on its plasma levels since creatinine is ultimately excreted into the urine. The plasma creatinine concentration tends not to be associated with FB exposure in piglets, as results are inconsistent. A 12.2 mg/kg diet for four weeks failed to induce the plasma creatinine level [45], whereas 6 and 8 mg FB/kg diets for five and four weeks, respectively, could elevate its concentration [21,52]. In contrast, a dose- and time-dependent increase at the creatinine level was shown in rats [53]. In our study, either creatinine clearance was lower in FB-fed animals (illustrated in male and female Swiss mice [54]), or its release into the plasma from the striated muscle was high. In summary, alterations in the nitrogenous compounds of blood plasma confirm slight/mild nephrotoxicity.

### 3.6. Plasma Lipid Metabolites and Glucose

Intergroup differences between FB and DZ treatments were tCHO and LDL after two weeks of exposure. FB down-regulates the liver X-nuclear receptors (LXR), a crucial mediator of cholesterol efflux and lipid homeostasis [29]. A recent study has proposed the disruption of LXR by FB as an alternative toxicity mode of action unrelated to the disruption of sphingolipid metabolism [55]. Elevated tCHO levels in blood serum induced by higher pure FB_1_ or FB doses was illustrated in swine [29,45,56,57,58,59], mice and rats [54] and in similar FB doses to those provided to our rabbits [20]. The key role of LDL is CHO transportation from the liver to other tissues. Thus, elevated tCHO was a repercussion of the high LDL level (positive association). High levels of tCHO and LDL in the FB group are attributed to the FB_1_-hepatotoxic mode of action [29,58], indicating a disruption in the lipoprotein endocytosis process of hepatocyte membranes. In the present study, the restoration of tCHO and LDL levels in the third week refers to the adaptive response and/or negligible hepatotoxicity that do not compromise their levels, probably due to the low dose applied.

### 3.7. Plasma Enzyme Activities

Fumonisins are known to induce liver injury/damage, whereas DON and ZEN are not distinctive hepatotoxins. No hepatic injury was proven in our study, underscored by the non-responsive enzyme activity values, such as LDH, ALT, AST and the AST/ALT ratio (hepatic injury biomarkers). This finding is consistent with the literature on the effects of low fusariotoxin doses on rabbits [20] and rats [16] but contrary to the findings of [15] in rats when FDZ increased ALT activity. Studies vary in model designs; unlike us, [16] and Szabó et al. [15] administrated pure fusariotoxins (non-fungal culture forms). Enzyme activities determined in the blood plasma revealed alterations in GGT and ALP activities in FB and FDZ fed groups, respectively. The effects of low doses of fusariotoxins on GGT and ALP are not being intensively investigated in the literature. None of them were responsive in rabbits [20], whereas the results in rats were inconsistent; Studies [15,16] have reported no response, whereas [22] showed an ALP increase in Sprague-Dawley male and female rats fed FDZ for 28 days.

GGT is a membrane-bound enzyme present in various tissues (including the liver and kidney) of all species, whereas the hepatic tissue is the most prominent [60]. GGT’s ultimate role is the regulation of the intracellular antioxidant glutathione [61]. In the present study, FB exposure for three weeks increased the GGT activity in blood plasma. Those findings are interesting, since a higher dose (8 mg FB/kg diet) for four weeks did not alter the GGT activity, neither in male nor female piglets [52]. Regardless of the breed-specific response and age differences, the discrepancy in GGT outcome might be attributed to the animal’s capacity for restoring homeostasis, which depends upon the organ injury severity. Even though this enzyme is the most sensitive indicator of hepatocellular damage or leakage, even the leakage of renal epithelial cells can increase plasma GGT activity. In our study, the hepato-histomorphological alterations were not present; a likely modification in GGT activity resulting from renal injury. The ALP activity was increased by the exposure of tertiary fusariotoxins, although no hepatic injury was proven (data not shown). The elevated ALP activities are probably attributed to four tissues; the pancreas, kidney, bone and intestinal tissues. Once hepato-pancreatoxicity was not attained, enterocyte damage and/or impairment in mineral balance are valid scenarios. We assumed that bone-mineral imbalance or mineral absorption was present since nephrotoxicity and alterations in P and Ca concentrations were confirmed. In our design, no enterocyte test was performed, but FCR was decreased, suggesting no implication in the piglet’s gastrointestinal tract. Thus, further investigations are necessary to assess fusariotoxins’ interactive effects on piglet GIT functionality.

### 3.8. Plasma Ion Concentrations

Effects of fusariotoxins on the plasma ion concentration are rarely discussed because their levels are associated with several conditions/organ functions. The non-altered Na concentration illustrates the short storage period for whole blood and the absence of significant dehydration in animals [62]. In our study, tertiary FDZ-administration for three weeks elevated the total and corrected Ca and P concentrations. An elevated P concentration was proven in male rats exposed to the tertiary combination of fusariotoxins for 14 days, but the Ca was not responsive [22]. Both ions are essential for survival, and the bone is their primary storage tissue. Ca is present in two forms; bound (mostly bound to albumin, whereas a minor portion is compounded with non-protein molecules) and unbound (it reflects the ionized form). The P is necessary for energy metabolism, nucleic acid and membrane lipid synthesis, and cellular signalling [63]. From the results, plasma proteins remained unchanged after FDZ exposure, suggestive of no loss in Ca complexes to proteins. Thus, the elevated total Ca-concentration was mainly a consequence of the increase in ionized-Ca concentration. Ionized-Ca serves numerous intracellular and extracellular roles, such as muscle contraction, neural signalling, enzymatic reaction, blood clot formation and cellular growth and division [62,63].

### 3.9. Antioxidant Enzymes and Lipid Peroxidation End Product

Fusariotoxins can induce oxidative stress, where the oxidation degree depends on the toxin; ZEN > DON > FB [64]. Among the investigated organs in our study, only the kidneys showed a response to FB exposure, lower MDA concentration and GPx activity. It can be concluded that other tissues have a more effective antioxidant system than does the kidney. Notably, FB exposure was potent in exerting its potential effect on the renal antioxidants. Our findings are contrary to findings in rats [15,16], which have shown that similar individual doses of FB/FB_1_ failed to alter the liver and kidney antioxidant systems.

The potency of FB in inducing oxidative stress has been well-reviewed and established, but ROS generation has been recognised as a consequence and not a direct toxicity mode of action [65]. MDA is the end product of the radical induced decomposition of polyunsaturated FA peroxidation and expresses cytotoxic effects [66]. Thus, as elevated by FB-exposure, MDA concentration was demonstrated in numerous animal models [15,34,67]. In our case, the MDA concentration decreased in the FB-group. The reduction in its concentration may occur because of the effective elimination of MDA by the antioxidant enzymatic system, the GPx. GPx is a family of enzymes homologous to selenocysteine (Sec); GPx1 is abundant in mammals [68]. This MDA decrease is probably achieved by activating the Nrf2/antioxidant response element (ARE) pathway [69], which exerts a cytoprotective mechanism. 

The GSH concentration was not responsive, although GPx activity was decreased. Besides an effective GSH synthesis in renal tissue, we assume some events might contribute to maintaining its level, such as the early stimulation of Nrf2/ARE (prior to minimal depletion of endogenous antioxidants) and alternative GPx members (does not need GSH) being involved in the ROS elimination. According to Labunskyy et al. [70], the GPx family comprises members that do not contain Sec at the active site nor use GSH. In summary, our data revealed the potency of FB at the permitted EU-feed limit in altering the renal antioxidant system. Furthermore, modifications induced by fusariotoxins in the polar lipid pools of the liver were independent of oxidative stress.

## 4. Conclusions

This is the first in vivo study reporting modulations in the membrane lipids of the kidney, liver and spleen from weaned piglets, as induced by combined fusariotoxin exposure at their permitted individual EU-guidance levels for the individual mycotoxins. Our data revealed that the applied fusariotoxins have negative impacts on kidney functionality (but not deteriorating glomerular filtration) and, to a lesser extent, on the liver (changing lipoprotein levels slightly), but are not harmful to the spleen. The profound effects of FB and FDZ on the membrane lipids might refer indirectly to ceramide synthesis inhibition or disturbance. Modifications in the blood plasma were recorded in the FB group, suggesting that further studies with low FB levels are necessary to verify NOAEL in swine (EFSA reported 1 mg FB/kg diet does not impose a significant change in pigs). Our study provides an LOAEL of 5 mg FB/kg diet for piglets, equivalent to 150 µg/kg body weight/day, which is similar to the proposed LOAEL (148 µg/kg body weight/day) [45]. However, neither clinical symptoms nor performance deterioration were found during the exposure period. The limitation of our study is the population size (six weaned piglets/treatment) and short exposure period (21 days), which may not allow for the observation of minor effects.

## 5. Materials and Methods

### 5.1. Experimental Animals and Diets

The study was carried out on 24 weaned borrowed piglets sharing the same genotype (Landrace X Yorkshire), at 50 days of age and 12–13 kg BWs. Animals were randomly grouped and assigned into 4 treatments/groups. Each treatment was composed of 6 piglets and only one piglet was kept in each pen (6 pens/6 piglets/treatment, i.e., individual pen allocation). Each pen consisted of a feeder attached to the feeder’s front side and an automatic drinker on the side. During the experiment, the feed was offered twice a day, and the remaining feed was measured to calculate the daily consumption. Drinking water was provided ad libitum throughout the experiment.

The experiment consisted of 4 treatments; control (mycotoxin-free), FB fed group, DON + ZEA (DZ) fed group, and FB + DON + ZEA (FDZ) fed group. In all treatments, animals were fed an identical toxin-free diet for seven days (an adaptation period), in which the nutrient composition of the diet corresponds to their age; proximate analysis can be seen in Table 11. The diet nutrients’ optimization was performed by Bonafarm-Bábolna Takarmány Kft (Bábolna, Hungary). After that, the other animals that were not in the control group, were orally fed mycotoxins through the diet for 21 days. Each diet was contaminated homogeneously with mycotoxins (FB, DZ and FDZ) fungal cultures; these were detected for each and the exact level was confirmed. For individual and mycotoxin mixtures, the applied doses were in accordance with the individual mycotoxin tolerance level in the feed [12], since the regulatory guidance for mycotoxin mixtures has not yet been established. The contaminated diets contained mycotoxins per each kilogram of diet as follows: FB fed group: 3.9 mg FB_1_ and a lower contamination with FB_2_ of 1.1 mg; DZ fed group: 0.9 mg DON and 0.1 mg ZEA; FDZ fed group: 5 mg FB, 0.9 mg DON and 0.1 ZEA. Generally, each diet was fed to its target animals to provide an approximate daily intake of 2.5 mg FB, 0.45 mg DON and 0.05 ZEA per animal/day (equivalent to 0.5 kg feed consumption/animal/day). 

### 5.2. Mycotoxin Production

*Fusarium verticillioides* (for FB culture material) and *Fusarium graminearum* (for DON at 28 °C and for ZEA a separate batch at 18 °C) (NRRL 20960 [MRC 826] and NRRL 5883, respectively) fungal culture (7 days old) was grown on 0.5 strength potato dextrose agar (PDA; Chemika-Biochemica, Basel, Switzerland). Agar discs (5 mm) were prepared with a cork borer (Boekel Scientifica, Feasterville, PA, USA), and were then stored at 10 °C in darkness in test tubes containing sterile distilled water (10 discs/10 mL).

For toxin production, maize (40 g) was soaked in distilled water (40 mL) at room temperature for 1 h in Erlenmeyer flasks (500 mL), closed with cotton wool plugs. This was followed by the addition of the inoculated agar discs (10 agar discs per flask) to the two-times autoclaved (20 min.) matrix. The cultures were then stored and incubated at 24 °C (FB), 28 °C (DON) and 18 °C (ZEA) for 3 weeks, thus having 3 culture materials. The flasks were shaken twice every day during the first week of incubation. When the incubation time was complete, the fungus-infected cereal was dried at room temperature and ground.

The homogenized fungal cultures contained FB_1_, FB_2_, DON and ZEA at concentrations of 3300, 930, 2010 and 1298 mg/kg, respectively. This stock material was included at its above-given concentration into the diets to reach the final target concentrations.

The concentration of the single and combined mycotoxins was as it was in the culture materials, so in the feeds it was determined with the LC-MS technique (LC-MS 2020, Shimadzu, Kyoto, Japan), as described earlier [71].

### 5.3. Sample Collections

Blood collection (in heparinized Vacutainer tubes, Fisher Scientific, Bishop Meadow Road, Loughborough, Leicestershire, UK) and BW measurement of individuals were performed on a weekly basis, whereas the feed intake was measured daily. Prior to sacrifice (12 h), the feed supply was stopped and only drinking water was available. At the end of the trial, the piglets were euthanized and exsanguinated after sedation (euthanyl-pentobarbital sodium, 240 mg/mL) and heart, liver, kidney, lung and spleen were weighed. The liver and kidney samples were collected and frozen immediately for further analysis to be performed.

### 5.4. Lipid Analysis

Kidney, liver and spleen samples (after storage at −20 °C) were homogenized separately (IKA T25 Digital Ultra Turrax, Staufen, Germany) in the 20-fold volume of chloroform-methanol (2:1, *v:v*) and the total lipid content was extracted according to [72]. Solvents were ultrapure-grade (Carl Roth GmbH + Co. KG, Karlsruhe, Germany) and 0.01% *w:v* butylated hydroxytoluene was added to prevent fatty acid oxidation.

For the separation of lipid fractions, namely the phospholipids, extracted total lipids were transferred to glass chromatographic columns containing 300 mg silica gel (230–400 mesh) for 10 mg of total lipids [73]. Neutral lipids were eluted with 10 mL chloroform for the above fat amount, then 15 mL acetone: methanol (9:1, *v/v*) was added, while 10 mL pure methanol eluted the total phospholipids. This latter fraction was evaporated under a nitrogen stream and was trans-methylated with a base-catalysed NaOCH_3_ method [74].

Fatty acid methyl esters were extracted into 300 μL ultrapure n-hexane for gas chromatography, which was performed on gas chromatography (Shimadzu Nexis 2030, Kyoto, Japan), equipped with a Phenomenex Zebron ZB-WAX Capillary GC column (30 m × 0.25 mm ID, 0.25 micrometre film, Phenomenex Inc., Torrance, CA, USA). Characteristic operating conditions were: injector temperature: 270 °C, helium flow: 28 cm/sec. The oven temperature was graded: from 80 to 205 °C: 2.5 °C/min, 5 min at 205 °C, from 205 to 250 °C 10 °C/min and 5 min at 210 °C. The makeup gas was nitrogen. To identify the individual FAs, an authentic external FA standard (37 Component FAME Mix, Merck Sigma-Aldrich, Cat. No.: CRM47885) was used. Fatty acid results were expressed as weight % of total fatty acid methyl esters.

The unsaturation index was defined as the number of double bonds in 100 fatty acyl chains. From the FA results, UI was calculated as follows: UI = [(1 × Σ monoenoic FA) + (2 × Σ dienoic FA) + (3 × Σ trienoic FA) + (4 × Σ tetraenoic FA) + (5 × Σ pentaenoic FA) + (6 × Σ hexaenoic FA)] [75]. The average fatty acyl chain length was calculated from the multiplication of the chain length values and the respective proportions of each FA.

### 5.5. Serum Clinical Chemistry Analysis

Blood samples were centrifuged at 1500 g immediately after collection for serum extraction. The different clinical parameters of serum nitrogenous compounds, lipid metabolites, enzyme activates, and ion concentrations were determined in a veterinary laboratory (Vet-Med Laboratory, Budapest, Hungary), using the Roche Hitachi 917 Chemistry Analyzer (Hitachi, Tokyo, Japan) with commercial diagnostic kits (Diagnosticum LTD., Budapest, Hungary).

### 5.6. Assessment of Antioxidant Capacity

For the determination of lipid peroxidation, blood plasma, kidney, liver and lung samples were stored at −80 °C until analysis. Lipid peroxidation was determined by quantifying malondialdehyde (MDA) levels by the 2-thiobarbituric acid method [76] in the blood plasma and 1:9 homogenates of tissue samples in physiological saline. Among the components of the antioxidant system, the concentration of reduced glutathione (GSH) was measured in the blood plasma and the 10,000 g supernatant fraction of tissue homogenates by the method of [77] and the activity of glutathione peroxidase (GPx) according to [78]. GSH concentration and GPx activity were calculated as protein content, determined by the biuret method in the blood plasma [79] and Folin phenol reagent in tissue homogenates [80].

### 5.7. Data Analysis

For the measured parameters (growth performance, lipid classes, clinical chemical parameters, antioxidant parameters, histopathological), an analysis of variance (ANOVA) was carried out using IBM Statistics software version 20 (2020). To identify the differences between the treatments, the post hoc Tukey’s multiple comparison test was used. The test was considered significant when the probability was lower than 0.05 (*p* < 0.05).

The Bliss independence method was applied to ascertain possible mycotoxin-treatment associated interactions in cases where there existed a significant difference between two or multiple groups [81]. It is based on the principle that drug effects are outcomes of probabilistic processes and assumes that drugs act independently so that neither of them interferes with the other (different sites/modes of action), but each contributes to a common result. The observed joint effect was expressed as a probability (0 ≤ E_AB_ ≤ 1) compared to the expected additive effect given by the common formula for probabilistic independence: E_A_ + E_B_ (1 − E_A_) = E_A_ + E_B_ − E_A_E_B_, where 0 ≤ E_A_ ≤ 1 and 0 ≤ E_B_ ≤ 1. Interaction types (additive, synergistic and antagonistic) were determined by comparing the effect probabilities of observed and expected joined effects. In the case of equal expected effect value to observed value, fusariotoxins’ interaction was additive, whereas, in higher and lower expected effect values, the fusariotoxins’ interactions were antagonistic and synergistic, respectively [82]. Only the significantly existing interaction results are shown in a textual form, merely for the cases where ANOVA also revealed significant inter-group differences.

Afterwards, Principal Component Analysis (PCA) was performed on the fatty acid profile of the different organs with the Unscrambler 9.7. software [83] to seek principal components describing the variance responsible for the “group formation” with the highest possible efficacy. The sole purpose of PCA was not to discriminate the certain groups of treatments based on their chemical composition, but to describe the basic orientation of the groups within the multidimensional space described by the variables investigated (e.g., FA profile). The orientation of the samples is described by the score plot showing the scores of each sample along with the first two principal components. The variable impact is presented with the loadings bar graph, which shows the contribution of the variance of each investigated variable to the variance of the first principal component, that is, the values of the loadings graph are the weights for each original variable when calculating the principal component.

## Figures and Tables

**Table 1:** Growth performance (BW: kg and AWG in kg/week), absolute (g) and relative (% of BW) organ weight in piglets fed experimental diets containing FB, DZ (DON + ZEA) and FDZ (FB + DON + ZEA) for 3 weeks, as compared to the control (results represent mean ± standard deviation).

Parameter	Control	FB	DZ	FDZ
	M ± SD	M ± SD	M ± SD	M ± SD
Initial BW	12.2 ± 0.61	12.8 ± 0.54	12.7 ± 0.67	12.4 ± 0.87
1-AWG	2.59 ± 0.34 ^c^	2.58 ± 0.20 ^b^	2.67 ± 0.24 ^b^	2.62 ± 0.21
2-AWG	1.98 ± 0.28 ^b^	1.95 ± 0.19 ^a^	1.98 ± 0.44 ^a^	2.22 ± 0.25
3-AWG	1.55 ± 0.23 ^Aa^	2.45 ± 0.41 ^bB^	2.40 ± 0.61 ^abB^	2.40 ± 0.92 ^B^
Final BW	18.3 ± 0.69 ^A^	19.8 ± 0.61 ^B^	19.7 ± 1.07 ^B^	19.7 ± 1.32 ^B^
Heart (g)	110 ± 13.0	110 ± 8.18	113 ± 6.47	107 ± 8.61
Liver (g)	406 ± 45.6 ^B^	398 ± 14.1 ^B^	379 ± 19.8 ^AB^	361 ± 23.3 ^A^
Kidney (g)	89.8 ± 5.97	93.4 ± 4.40	85.6 ± 8.10	92.7 ± 16.9
Lung (g)	226 ± 55.8	223 ± 31.3	225 ± 46.4	201 ± 44.8
Spleen (g)	35.0 ± 5.14	36.4 ± 2.26	36.1 ± 5.91	36.1 ± 5.61
Rel. heart (%)	0.60 ± 0.05	0.56 ± 0.04	0.57 ± 0.03	0.55 ± 0.04
Rel. liver (%)	2.22 ± 0.24 ^B^	2.01 ± 0.05 ^AB^	1.93 ± 0.11 ^A^	1.85 ± 0.18 ^A^
Rel. kidney (%)	0.49 ± 0.03	0.47 ± 0.03	0.44 ± 0.05	0.47 ± 0.07
Rel. lung (%)	1.24 ± 0.32	1.13 ± 0.15	1.15 ± 0.27	1.01 ± 0.16
Rel. spleen (%)	0.19 ± 0.03	0.19 ± 0.01	0.18 ± 0.03	0.19 ± 0.03

BW, bodyweight, 1, 2 and 3, week number; AWG, Average bodyweight gain; Rel., relative; ^A,B, AB^, values with different uppercase capital letters within the same row are differences between treatments (*p* < 0.05); ^a,b,c,ab^, values with different uppercase small letters within the same column are differences between periods (*p* < 0.05).

**Table 2:** Total and weekly feeding parameters (g) of piglets fed experimental diets containing FB, DZ (DON + ZEA) and FDZ (FB + DON + ZEA) for 3 weeks, as compared to the control (results represent mean ± standard deviation).

Parameter	Period	Control	FB	DZ	FDZ
		M ± SD	M ± SD	M ± SD	M ± SD
FC (g/week)	overall	12,201 ± 469	12,463 ± 89.4	12,492 ± 19.6	12,499 ± 1.71
	1-week	3930 ± 414	4063 ± 89.4 ^a^	4092 ± 19.6 ^a^	4099 ± 1.71 ^a^
	2-week	4163 ± 91.3	4200 ± 0.00 ^b^	4200 ± 0.00 ^b^	4200 ± 0.00 ^b^
	3-week	4108 ± 225	4200 ± 0.00 ^b^	4200 ± 0.00 ^b^	4200 ± 0.00 ^b^
FCR (g diet/g BW)	overall	2.00 ± 0.08 ^A^	1.79 ± 0.10 ^B^	1.78 ± 0.14 ^B^	1.75 ± 0.19 ^B^
	1-week	1.53 ± 0.06 ^c^	1.58 ± 0.10 ^b^	1.54 ± 0.14 ^b^	1.58 ± 0.15
	2-week	2.13 ± 0.30 ^b^	2.17 ± 0.19 ^a^	2.23 ± 0.65 ^a^	1.91 ± 0.19
	3-week	2.69 ± 0.34 ^aA^	1.76 ± 0.33 ^bB^	1.84 ± 0.41 ^abB^	1.91 ± 0.53 ^B^

FC, feed consumption; FCR, feed conversion ratio; ^A,B, AB^, values with different uppercase capital letters within the same row are differences between treatments (*p* < 0.05); ^a,b,c,ab^, values with different uppercase small letters within the same column are different between periods (*p* < 0.05).

**Table 3:** Fatty acid profile of the renal total phospholipids of piglets fed experimental diets containing FB, DZ (DON + ZEA) and FDZ (FB + DON + ZEA) for 3 weeks (as compared to the control); results represent mean ± standard deviation.

Fatty Acid	Control	FB	DZ	FDZ
	M ± SD	M ± SD	M ± SD	M ± SD
C14:0	0.14 ± 0.02 ^a^	0.17 ± 0.02 ^b^	0.14 ± 0.01 ^ab^	0.17 ± 0.02 ^ab^
C16:0	16.2 ± 0.76 ^a^	17.4 ± 0.59 ^b^	16.8 ± 0.42 ^ab^	17. 7 ± 0.48 ^b^
C16:1	0.27 ± 0.03 ^a^	0.32 ± 0.04 ^ab^	0.33 ± 0.03 ^ab^	0.33 ± 0.03 ^b^
C18:0	19.1 ± 0.57	19.4 ± 0.57	19.2 ± 0.21	19.5 ± 0.25
C18:1n9	12.5 ± 0.40	12.1 ± 0.64	11.9 ± 0.64	12.6 ± 0.46
C18:1n7	1.99 ± 0.05	2.03 ± 0.20	2.15 ± 0.14	2.09 ± 0.15
C18:2n6	17.9 ± 0.91	18.6 ± 0.72	18.6 ± 0.41	18.3 ± 0.49
C18:3n6	0.08 ± 0.01	0.09 ± 0.01	0.08 ± 0.01	0.10 ± 0.02
C18:3n3	0.31 ± 0.14	0.39 ± 0.03	0.38 ± 0.03	0.30 ± 0.17
C18:4n3	0.11 ± 0.13	0.05 ± 0.01	0.03 ± 0.02	0.10 ± 0.15
C20:0	0.12 ± 0.03 ^b^	0.07 ± 0.01 ^a^	0.08 ± 0.01 ^a^	0.09 ± 0.01 ^a^
C20:1n9	0.25 ± 0.05	0.22 ± 0.04	0.23 ± 0.03	0.22 ± 0.01
C20:2n6	0.75 ± 0.06 ^b^	0.71 ± 0.03 ^ab^	0.70 ± 0.08 ^ab^	0.63 ± 0.05 ^a^
C20:3n9	0.09 ± 0.01	0.09 ± 0.01	0.09 ± 0.00	0.09 ± 0.01
C20:3n6	1.11 ± 0.07	1.23 ± 0.14	1.17 ± 0.09	1.27 ± 0.26
C20:4n6	17.2 ± 0.85	16.7 ± 1.23	17.5 ± 0.63	16.6 ± 0.89
C20:3n3	0.20 ± 0.02 ^b^	0.18 ± 0.03 ^ab^	0.19 ± 0.03 ^ab^	0.16 ± 0.01 ^a^
C20:4n3	0.09 ± 0.01	0.11 ± 0.03	0.10 ± 0.01	0.11 ± 0.04
C20:5n3	7.23 ± 0.52	6.34 ± 0.47	6.61 ± 0.72	6.50 ± 0.59
C22:0	0.18 ± 0.07 ^b^	0.07 ± 0.03 ^a^	0.10 ± 0.01 ^a^	0.07 ± 0.01 ^a^
C22:1n9	0.04 ± 0.01 ^b^	0.03 ± 0.01 ^a^	0.04 ± 0.01 ^ab^	0.03 ± 0.01 ^a^
C22:5n3	1.45 ± 0.15 ^b^	1.47 ± 0.22 ^b^	1.23 ± 0.04 ^ab^	1.14 ± 0.09 ^a^
C24:0	0.12 ± 0.02 ^b^	0.09 ± 0.04 ^ab^	0.06 ± 0.03 ^a^	0.07 ± 0.03 ^ab^
C22:6n3	2.60 ± 0.25 ^b^	2.18 ± 0.17 ^a^	2.30 ± 0.15 ^ab^	2.24 ± 0.30 ^ab^
SFA	35.8 ± 0.31 ^a^	37.3 ± 0.54 ^b^	36.4 ± 0.29 ^a^	37.5 ± 0.66 ^b^
UFA	64.2 ± 0.31 ^b^	62.7 ± 0.54 ^a^	63.6 ± 0.29 ^b^	62.5 ± 0.66 ^a^
MUFA	15.1 ± 0.49	14.8 ± 0.91	14.8 ± 0.77	15.3 ± 0.59
PUFA	49.2 ± 0.52 ^b^	48.1 ± 0.82 ^ab^	49.0 ± 0.78 ^b^	47.3 ± 0.75 ^a^
PUFA/MUFA	3.26 ± 0.13	3.27 ± 0.25	3.32 ± 0.21	3.10 ± 0.15
n-3	12.0 ± 0.43 ^b^	10.7 ± 0.51 ^a^	10.8 ± 0.73 ^a^	10.3 ± 0.85 ^a^
n-6	37.1 ± 0.55 ^ab^	37.3 ± 0.89 ^ab^	38.0 ± 0.40 ^b^	36.9 ± 0.48 ^a^
n-6/n-3	3.10 ± 0.14 ^a^	3.49 ± 0.22 ^ab^	3.52 ± 0.26 ^b^	3.59 ± 0.32 ^b^
UI	183 ± 1.95 ^c^	176 ± 3.09 ^ab^	179 ± 3.19 ^bc^	173 ± 1.94 ^a^
ACL	19.0 ± 0.19 ^b^	18.6 ± 0.29 ^ab^	18.4 ± 0.18 ^a^	18.4 ± 0.21 ^a^

^a,b,c,^ different small uppercase indices mean significant difference (*p* < 0.05); SFA, Saturated fatty acids; UFA, Unsaturated fatty acids, MUFA, Monounsaturated fatty acids; PUFA, Polyunsaturated fatty acids; n-3, Omega-3; n-6, Omega-6; n-6/n-3, Omega-6:Omega-3; UI, Unsaturation index; ACL, Average chain length.

**Table 4:** Fatty acid profile of the total phospholipids from the hepatic tissue for piglets fed experimental diets containing FB, DZ (DON + ZEA) and FDZ (FB + DON + ZEA) for 3 weeks (as compared to the control); results represent mean ± standard deviation.

Fatty Acid	Control	FB	DZ	FDZ
	M ± SD	M ± SD	M ± SD	M ± SD
C14:0	0.07 ± 0.01 ^a^	0.10 ± 0.01 ^b^	0.08 ± 0.01 ^ab^	0.08 ± 0.01 ^ab^
C16:0	13.0 ± 0.63	13.2 ± 0.47	13.6 ± 0.55	13.3 ± 0.15
C16:1	0.32 ± 0.04 ^a^	0.41 ± 0.04 ^b^	0.40 ± 0.04 ^b^	0.37 ± 0.04 ^ab^
C18:0	29.4 ± 0.80 ^b^	28.1 ± 0.86 ^a^	28.1 ± 0.87 ^ab^	29.2 ± 0.38 ^ab^
C18:1n9	7.05 ± 0.46 ^a^	8.28 ± 0.34 ^b^	7.58 ± 0.45 ^ab^	7.71 ± 0.55 ^ab^
C18:1n7	1.31 ± 0.12	1.64 ± 0.28	1.57 ± 0.29	1.37 ± 0.14
C18:2n6	14.6 ± 0.92	15.5 ± 0.68	14.5 ± 0.81	14.3 ± 0.44
C18:3n6	0.13 ± 0.02	0.15 ± 0.04	0.15 ± 0.03	0.16 ± 0.04
C18:3n3	0.21 ± 0.05	0.26 ± 0.05	0.21 ± 0.03	0.24 ± 0.03
C18:4n3	0.03 ± 0.02 ^ab^	0.05 ± 0.03 ^b^	0.02 ± 0.01 ^a^	0.02 ± 0.00 ^ab^
C20:0	0.04 ± 0.01	0.05 ± 0.01	0.04 ± 0.01	0.04 ± 0.00
C20:1n9	0.12 ± 0.01	0.14 ± 0.01	0.12 ± 0.02	0.12 ± 0.01
C20:2n6	0.42 ± 0.06	0.46 ± 0.03	0.42 ± 0.09	0.41 ± 0.05
C20:3n9	0.12 ± 0.08	0.13 ± 0.04	0.16 ± 0.04	0.12 ± 0.02
C20:3n6	0.73 ± 0.04	0.69 ± 0.13	0.73 ± 0.15	0.83 ± 0.13
C20:4n6	16.4 ± 0.81	16.4 ± 0.60	16.8 ± 0.82	16.2 ± 0.69
C20:3n3	0.05 ± 0.01	0.06 ± 0.01	0.05 ± 0.02	0.05 ± 0.01
C20:4n3	0.02 ± 0.00	0.02 ± 0.00	0.02 ± 0.00	0.02 ± 0.00
C20:5n3	3.94 ± 0.31	3.72 ± 0.24	3.46 ± 0.48	3.66 ± 0.44
C22:0	0.05 ± 0.01 ^b^	0.03 ± 0.01 ^ab^	0.02 ± 0.00 ^a^	0.02 ± 0.00 ^a^
C22:5n3	2.46 ± 0.33	2.63 ± 0.37	2.62 ± 0.20	2.48 ± 0.48
C24:0	0.02 ± 0.01	0.02 ± 0.01	0.01 ± 0.00	0.01 ± 0.00
C22:6n3	9.64 ± 0.75 ^b^	8.02 ± 0.61 ^a^	9.25 ± 0.42 ^b^	9.32 ± 0.44 ^b^
SFA	42.5 ± 0.48 ^b^	41.4 ± 0.59 ^a^	41.9 ± 0.64 ^ab^	42.6 ± 0.48 ^b^
UFA	57.5 ± 0.48 ^a^	58.6 ± 0.59 ^b^	58.1 ± 0.64 ^ab^	57.4 ± 0.48 ^a^
MUFA	8.80 ± 0.53 ^a^	10.5 ± 0.35 ^b^	9.67 ± 0.62 ^ab^	9.57 ± 0.67 ^ab^
PUFA	48.7 ± 0.66	48.1 ± 0.51	48.5 ± 0.50	47.8 ± 0.49
PUFA/MUFA	5.55 ± 0.38 ^b^	4.60 ± 0.17 ^a^	5.03 ± 0.35 ^ab^	5.02 ± 0.37 ^ab^
n-3	16.3 ± 1.04 ^b^	14.8 ± 1.11 ^a^	15.6 ± 0.43 ^ab^	15.8 ± 0.63 ^ab^
n-6	32.2 ± 1.00	33.2 ± 0.98	32.7 ± 0.64	31.9 ± 0.75
n-6/n-3	1.98 ± 0.18	2.27 ± 0.25	2.09 ± 0.09	2.02 ± 0.12
UI	198 ± 3.86 ^b^	192 ± 4.06 ^a^	197 ± 2.08 ^ab^	195 ± 1.98 ^ab^
ACL	18.7 ± 0.03 ^b^	18.6 ± 0.03 ^a^	18.6 ± 0.02 ^ab^	18.6 ± 0.03 ^ab^

^a,b,c,^ different small uppercase indices mean significant difference (*p* < 0.05); SFA, Saturated fatty acids; UFA, Unsaturated fatty acids, MUFA, Monounsaturated fatty acids; PUFA, Polyunsaturated fatty acids; n-3, Omega-3; n-6, Omega-6; n-6/n-3, Omega-6:Omega-3; UI, Unsaturation index; ACL, Average chain length.

**Table 5:** Fatty acid profile of the total phospholipids from the spleen for piglets fed experimental diets containing FB, DZ (DON + ZEA) and FDZ (FB + DON + ZEA) for 3 weeks (as compared to the control); results represent mean ± standard deviation.

Fatty Acid	Control	FB	DZ	FDZ
	M ± SD	M ± SD	M ± SD	M ± SD
C14:0	0.34 ± 0.03	0.33 ± 0.08	0.31 ± 0.02	0.34 ± 0.03
C14:1	0.00 ± 0.00	0.00 ± 0.00	0.00 ± 0.00	0.01 ± 0.01
C16:0	22.9 ± 0.72	23.0 ± 1.41	22.4 ± 0.42	22.9 ± 0.56
C16:1	0.41 ± 0.02	0.40 ± 0.04	0.41 ± 0.03	0.41 ± 0.04
C18:0	19.7 ± 0.54	19.6 ± 0.87	19.8 ± 0.40	19.5 ± 0.74
C18:1n9	10.6 ± 0.40	10.7 ± 0.61	10.4 ± 0.37	10.9 ± 0.23
C18:1n7	2.15 ± 0.09	2.13 ± 0.15	2.31 ± 0.11	2.17 ± 0.15
C18:2n6	10.6 ± 0.78	10.5 ± 0.46	9.89 ± 0.83	10.0 ± 0.64
C18:3n6	0.17 ± 0.03	0.17 ± 0.02	0.18 ± 0.04	0.19 ± 0.01
C18:3n3	0.10 ± 0.02	0.12 ± 0.01	0.11 ± 0.01	0.12 ± 0.02
C18:4n3	0.08 ± 0.01 ^a^	0.11 ± 0.05 ^ab^	0.13 ± 0.04 ^ab^	0.18 ± 0.11 ^b^
C20:0	0.13 ± 0.01 ^ab^	0.12 ± 0.01 ^a^	0.14 ± 0.20 ^b^	0.28 ± 0.02 ^ab^
C20:1n9	0.30 ± 0.04	0.28 ± 0.02	0.30 ± 0.02	0.28 ± 0.02
C20:2n6	0.81 ± 0.10	0.84 ± 0.06	0.83 ± 0.10	0.84 ± 0.06
C20:3n9	0.10 ± 0.03	0.09 ± 0.02	0.11 ± 0.03	0.10 ± 0.01
C20:3n6	2.08 ± 0.12	2.08 ± 0.13	2.05 ± 0.11	2.13 ± 0.19
C20:4n6	20.1 ± 1.23	20.6 ± 0.61	21.3 ± 0.90	20.7 ± 0.60
C20:3n3	0.08 ± 0.01	0.09 ± 0.01	0.08 ± 0.01	0.10 ± 0.01
C20:4n3	0.03 ± 0.01	0.04 ± 0.00	0.03 ± 0.00	0.04 ± 0.01
C20:5n3	2.01 ± 0.13	1.91 ± 0.15	1.77 ± 0.21	1.94 ± 0.25
C22:0	0.13 ± 0.01 ^bc^	0.10 ± 0.02 ^ab^	0.14 ± 0.02 ^c^	0.09 ±0.02 ^a^
C22:1n9	0.08 ± 0.02	0.07 ± 0.01	0.08 ± 0.01	0.08 ± 0.01
C22:2n6	0.04 ± 0.01	0.04 ± 0.00	0.04 ± 0.01	0.04 ± 0.00
C22:5n3	3.83 ± 0.06	3.64 ± 0.20	3.87 ± 0.37	3.64 ± 0.11
C24:0	0.05 ± 0.01 ^ab^	0.05 ± 0.03 ^ab^	0.06 ± 0.03 ^b^	0.03 ± 0.01 ^a^
C22:6n3	3.23 ± 0.16	2.98 ± 0.18	3.26 ± 0.22	3.20 ± 0.24
SFA	43.2 ± 0.36	43.2 ± 0.62	42.7 ± 0.55	42.9 ± 1.02
UFA	56.8 ± 0.36	56.8 ± 0.62	57.1 ± 0.55	57.1 ± 1.02
MUFA	13.6 ± 0.51	13.6 ± 0.62	13.5 ± 0.42	13.8 ± 0.37
PUFA	43.2 ± 0.51	43.2 ± 0.76	43.6 ± 0.40	43.2 ± 0.70
PUFA/MUFA	3.19 ± 0.15	3.19 ± 0.18	3.22 ± 0.11	3.13 ± 0.05
n-3	9.37 ± 0.12 ^b^	8.88 ± 0.27 ^a^	9.25 ± 0.30 ^b^	9.21 ± 0.45 ^ab^
n-6	33.7 ± 0.48	34.3 ± 0.80	34.2 ± 0.30	33.9 ± 0.47
n-6/n-3	3.60 ± 0.06 ^a^	3.86 ± 0.17 ^b^	3.70 ± 0.13 ^ab^	3.69 ± 0.18 ^ab^
UI	173 ± 3.34	172 ± 2.79	176 ± 1.59	174 ± 2.17
ACL	18.3 ± 0.04 ^ab^	18.3 ± 0.05 ^a^	18.4 ± 0.01 ^b^	18.3 ± 0.01 ^ab^

^a,b,c,^ different small uppercase indices mean significant difference (*p* < 0.05); SFA, Saturated fatty acids; UFA, Unsaturated fatty acids, MUFA, Monounsaturated fatty acids; PUFA, Polyunsaturated fatty acids; n:3, Omega-3; n:6, Omega-6; n:6/n:3, Omega-6:Omega-3; UI, Unsaturation index; ACL, Average chain length.

**Table 6:** The plasma nitrogenous compounds in a weekly assessment of piglets fed experimental diets containing FB, DZ (DON + ZEA) and FDZ (FB + DON + ZEA) for 3 weeks, as compared to the control); results represent mean ± standard deviation.

Parameter	Period	Control	FB	DZ	FDZ
		M ± SD	M ± SD	M ± SD	M ± SD
Total Protein (g/L)	1-week	53.2 ± 1.33 ^a^	52.2 ± 2.86	50.8 ± 3.87	52.8 ± 3.54 ^a^
	2-week	55.0 ± 2.19 ^ab^	55.5 ± 2.59	53.2 ± 3.54	57.8 ± 3.60 ^b^
	3-week	55.8 ± 1.60 ^b^	56.0 ± 3.52	54.4 ± 4.22	58.0 ± 2.97 ^b^
Albumin (g/L)	1-week	37.3 ± 2.50	35.3 ± 1.86	35.3 ± 3.93	35.8 ± 4.31
	2-week	39.7 ± 3.20	37.5 ± 2.26	38.5 ± 3.73	38.7 ± 3.44
	3-week	41.0 ± 3.46	37.8 ± 2.71	38.8 ± 3.27	39.2 ± 3.54
Urea (mmol/L)	1-week	2.87 ± 0.27 ^AbB^	3.34 ± 0.21^B^	2.83 ± 0.42 ^A^	2.50 ± 0.18 ^A^
	2-week	3.25 ± 0.63 ^b^	3.05 ± 0.40	3.02 ± 0.50	2.62 ± 0.33
	3-week	2.05 ± 0.32 ^aA^	2.88 ± 0.23 ^B^	2.78 ± 0.20 ^B^	2.28 ± 0.35 ^A^
Creatinine (μmol/L)	1-week	75.6 ± 8.65 ^aA^	93.5 ± 10.4 ^B^	80.3 ± 11.1 ^AB^	77.7 ± 8.16 ^aAB^
	2-week	87.7 ± 9.27 ^Aab^	107 ± 10.8 ^B^	94.7 ± 10.5 ^AB^	92.2 ± 5.45 ^AbB^
	3-week	88.0 ± 7.10 ^Ab^	112 ± 17.0 ^B^	94.0 ± 12.0 ^AB^	102 ± 10.9 ^AbB^

^A,B, AB^, values with different uppercase capital letters within the same row are differences between treatments (*p* < 0.05); ^a,b,ab,c^ values with different uppercase small letters within the same column are differences between periods (*p* < 0.05).

**Table 7:** The weekly data of plasma glucose and lipid metabolites (mmol/L) in piglets fed experimental diets containing FB, DZ (DON+ZEA) and FDZ (FB+DON+ZEA) for 3 weeks (as compared to the control); results represent mean ± standard deviation.

Parameter	Period	Control	FB	DZ	FDZ
		M ± SD	M ± SD	M ± SD	M ± SD
Glucose	1-week	6.02 ± 0.63 ^b^	6.84 ± 0.61 ^b^	6.03 ± 0.40 ^b^	6.52 ± 1.03 ^b^
	2-week	6.05 ± 0.57 ^b^	7.15 ± 0.74 ^b^	6.88 ± 0.81 ^b^	6.65 ± 0.56 ^b^
	3-week	4.12 ± 0.46 ^a^	4.22 ± 0.93 ^a^	4.08 ± 0.60 ^a^	4.18 ± 0.68 ^a^
TGs	1-week	0.43 ± 0.06 ^a^	0.53 ± 0.10	0.47 ± 0.09	0.44 ± 0.11
	2-week	0.55 ± 0.09 ^b^	0.58 ± 0.11	0.49 ± 0.10	0.56 ± 0.21
	3-week	0.37 ± 0.07 ^a^	0.47 ± 0.07	0.43 ± 0.12	0.47 ± 0.10
tCHO	1-week	2.34 ± 0.19	2.44 ± 0.25	2.16 ± 0.23	2.31 ± 0.28
	2-week	2.37 ± 0.16 ^AB^	2.65 ± 0.24 ^B^	2.25 ± 0.16 ^A^	2.51 ± 0.28 ^AB^
	3-week	2.38 ± 0.16	2.48 ± 0.24	2.31 ± 0.09	2.53 ± 0.25
HDL	1-week	1.09 ± 0.12	1.08 ± 0.16	1.03 ± 0.14	1.07 ± 0.23
	2-week	1.12 ± 0.12	1.20 ± 0.11	1.14 ± 0.12	1.20 ± 0.19
	3-week	1.14 ± 0.16	1.09 ± 0.15	1.16 ± 0.11	1.21 ± 0.18
LDL	1-week	1.06 ± 0.14	1.12 ± 0.10	0.91 ± 0.12	1.04 ± 0.20
	2-week	0.99 ± 0.09 ^AB^	1.18 ± 0.17 ^B^	0.89 ± 0.12 ^A^	1.05 ± 0.14 ^AB^
	3-week	1.09 ± 0.06	1.18 ± 0.19	0.95 ± 0.11	1.10 ± 0.26

TGs, triglycerides; CHO, cholesterol; HDL, high density lipoprotein; LDL, low density lipoprotein; ^A,B, AB^, values with different uppercase capital letters within the same row are differences between treatments (*p* < 0.05); ^a,b,ab^, values with different uppercase small letters within the same row are differences between periods (*p* < 0.05).

**Table 8:** The weekly plasma enzyme activities (in all cases IU/L) in piglets fed diets experimental containing FB, DZ (DON + ZEA) and FDZ (FB + DON + ZEA) for 3 weeks (as compared to the control); results represent mean ± standard deviation.

Parameter	Period	Control	FB	DZ	FDZ
		M ± SD	M ± SD	M ± SD	M ± SD
LDH	1-week	1093 ± 209	1009 ± 132 ^a^	1101 ± 281	1055 ± 208
	2-week	1093 ± 186	1163 ± 157 ^ab^	972 ± 78.0	1007 ± 137
	3-week	1059 ± 174	1237 ± 147 ^b^	1263 ± 363	1097 ± 118
AST	1-week	96.7 ± 51.1	53.8 ± 15.5	66.2 ± 42.6	62.5 ± 21.8
	2-week	66.5 ± 29.9	68.0 ± 27.3	40.8 ± 7.55	68.2 ± 38.8
	3-week	60.7 ± 25.7	72.8 ± 19.7	79.0 ± 38.4	56.8 ± 10.9
ALT	1-week	70.8 ± 11.0	78.7 ± 18.8	67.8 ± 17.4	71.5 ± 16.0
	2-week	72.8 ± 13.5	89.8 ± 22.9	73.0 ± 18.8	78.0 ± 20.0
	3-week	70.8 ± 16.4	81.3 ± 14.9	74.0 ± 14.9	70.2 ± 15.1
AST/ALT	1-week	1.35 ± 0.63	0.70 ± 0.19	1.00 ± 0.59	0.87 ± 0.23
	2-week	1.00 ± 0.72	0.74 ± 0.13	0.57 ± 0.07	0.85 ± 0.29
	3-week	0.89 ± 0.44	0.90 ± 0.18	0.99 ± 0.55	0.83 ± 0.16
GGT	1-week	32.7 ± 9.93	48.0 ± 11.5	36.0 ± 15.9	45.8 ± 8.98
	2-week	32.7 ± 12.6 ^A^	52.8 ± 12.9 ^AB^	36.0 ± 12.9 ^A^	57.7 ± 25.1 ^B^
	3-week	38.3 ± 14.4 ^A^	70.0 ± 16.6 ^B^	54.6 ± 20.4 ^AB^	64.5 ± 14.9 ^AB^
ALP	1-week	359 ± 53.9	412 ± 84.5	339 ± 70.3	401 ± 134
	2-week	327 ± 59.1	378 ± 87.9	284 ± 44.7	386 ± 122
	3-week	284 ± 47.6 ^AB^	334 ± 69.6 ^AB^	264 ± 64.2 ^A^	373 ± 102 ^B^
CK	1-week	1318 ± 668	718 ± 177	1064 ± 294	1551 ± 599
	2-week	1093 ± 462	976 ± 485	987 ± 409	1038 ± 492
	3-week	1225 ± 412	1044 ± 389	1479 ± 536	1097 ± 533

LDH, Lactate dehydrogenase; AST, Aspartate transaminase, ALT, Alanine transaminase, GGT, Gamma-glutamyltransferase; ALP, Alkaline phosphatase, CK, Creatine kinase; ^A,B, AB^, values with different uppercase capital letters within the same row are differences between treatments (*p* < 0.05); ^a,b,ab^, values with different uppercase small letters within the same row are differences between periods (*p* < 0.05).

**Table 9:** The plasma weekly ion levels (in all cases mmol/L) in piglets fed experimental diets containing FB, DZ (DON + ZEA) and FDZ (FB + DON + ZEA) for 3 weeks (as compared to the control); results represent mean ± standard deviation.

Parameter	Period	Control	FB	DZ	FDZ
		M ± SD	M ± SD	M ± SD	M ± SD
Na	1-week	147 ± 2.51	150 ± 2.42	148 ± 1.60	148 ± 1.87
	2-week	149 ± 2.34	149 ± 2.50	149 ± 1.97	149 ± 2.37
	3-week	148 ± 1.94	149 ± 1.05	148 ± 1.67	149 ± 1.86
Ca	1-week	3.23 ± 0.11 ^b^	3.41 ± 0.14 ^b^	3.20 ± 0.21 ^b^	3.22 ± 0.27
	2-week	3.18 ± 0.17 ^b^	3.11 ± 0.27 ^b^	3.22 ± 0.12 ^b^	3.13 ± 0.28
	3-week	2.54 ± 0.23 ^aA^	2.65 ± 0.18 ^aAB^	2.49 ± 0.33 ^aA^	2.85 ± 0.27 ^B^
Corr. Ca	1-week	2.05 ± 0.08 ^b^	2.23 ± 0.11 ^c^	2.16 ± 0.39 ^b^	2.06 ± 0.19 ^b^
	2-week	1.96 ± 0.17 ^b^	1.94 ± 0.23 ^b^	2.02 ± 0.12 ^b^	1.94 ± 0.19 ^ab^
	3-week	1.40 ± 0.22 ^aA^	1.56 ± 0.19 ^aAB^	1.40 ± 0.27 ^aA^	1.69 ± 0.23 ^aB^
P	1-week	2.85 ± 0.17 ^B^	2.63 ± 0.11 ^aAB^	2.51 ± 0.12 ^aA^	2.56 ± 0.25 ^aA^
	2-week	2.67 ± 0.20 ^b^	2.66 ± 0.13 ^a^	2.45 ± 0.08 ^a^	2.75 ± 0.46 ^ab^
	3-week	2.62 ± 0.12 ^A^	3.10 ± 0.27 ^AbB^	3.15 ± 0.43 ^AbB^	3.07 ± 0.22 ^bB^

Na, sodium; K, potassium; Ca, calcium; Corr Ca, corrected calcium; P, phosphorus; ^A,B, AB^, values with different uppercase capital letters within the same row are differences between treatments (*p* < 0.05); ^a,b,ab^, values with different uppercase small letters within the same column are differences between periods (*p* < 0.05).

**Table 10:** The plasma antioxidant capacity on a weekly basis and the kidney, liver and lung data after slaughter of piglets fed experimental diets containing FB, DZ (DON + ZEA) and FDZ (FB + DON + ZEA) for 3 weeks (as compared to the control); results represent mean ± standard deviation. (GSH: micromol/g protein.

Plasma					
Parameter	Period	Control	FB	DZ	FDZ
		M ± SD	M ± SD	M ± SD	M ± SD
GSH (micromol/g prot.)	1-week	2.88 ± 0.24	2.94 ± 0.30	2.86 ± 0.20 ^a^	2.88± 0.26
	2-week	2.68 ± 0.77	2.62 ± 0.49	2.31 ± 0.66 ^ab^	2.87 ± 0.38
	3-week	3.15 ± 0.18	3.10 ± 0.26	3.34 ± 0.13 ^b^	2.98 ± 0.33
GPx (U/g prot.)	1-week	3.29 ± 0.35	3.25 ± 0.32 ^a^	3.36 ± 0.52 ^a^	3.47 ± 0.27
	2-week	3.49 ± 0.29	3.28 ± 0.17 ^a^	3.16 ± 0.17 ^a^	3.22 ± 0.26
	3-week	4.07 ± 0.96	3.81 ± 0.34 ^b^	3.93 ± 0.15 ^b^	4.07 ± 0.92
MDA (nmol/g)	1-week	7.21 ± 1.10	8.13 ± 0.48	7.56 ± 1.22	7.76 ± 0.95
	2-week	8.56 ± 1.15	8.86 ± 1.09	7.65 ± 0.97	8.72 ± 1.43
	3-week	8.31 ± 0.33	9.20 ± 0.80	8.22 ± 0.76	8.75 ± 0.52
**Kidney**					
GSH (micromol/g prot.)	3-week	3.91 ± 0.46	3.05 ± 0.57	3.84 ± 0.63	3.14 ± 0.96
GPx (U/g prot.)	3-week	4.28 ± 0.65 ^B^	3.35 ± 0.51 ^A^	4.09 ± 0.67 ^AB^	3.19 ± 0.34 ^A^
MDA(nmol/g)	3-week	46.9 ± 6.43 ^B^	35.2 ± 4.54 ^A^	43.8 ± 8.51 ^AB^	42.5 ± 6.04 ^AB^
**Liver**					
GSH (micromol/g prot.)	3-week	5.30 ± 0.88	4.59 ± 0.50	4.13 ± 0.21	4.59 ± 1.04
GPx (U/g prot.)	3-week	4.63 ± 0.70	4.33 ± 0.62	4.10 ± 0.34	4.27 ± 1.05
MDA(nmol/g)	3-week	47.0 ± 7.87	47.2 ± 8.14	43.1 ± 14.3	50.0 ±13.3
**Lung**					
GSH (micromol/g prot.)	3-week	2.92 ± 0.41	2.66 ± 0.66	2.57 ± 0.57	2.66± 0.40
GPx (U/g prot.)	3-week	3.38 ± 0.50	3.06 ± 0.29	2.97 ± 0.49	3.04 ± 0.17
MDA(nmol/g)	3-week	28.6 ± 5.18	32.2 ± 5.71	31.5 ± 4.14	32.5 ± 4.38

GSH, reduced glutathione; GSHPx, glutathione peroxidase; MDA, malondialdehyde; ^A,B, AB^, values with different uppercase capital letters within the same row are differences between treatments (*p* < 0.05); ^a,b,ab^, values with different uppercase small letters within the same column are differences between periods (*p* < 0.05).

**Table 11:** The diet composition of piglets during the study period (3 weeks).

Composition	Calculated from 100% Dry Matter/kg
DE (MJ/kg)	16.3
ME (MJ/kg)	15.6
CP (%)	19.8
Lysine (%)	1.5
Methionine (%)	0.6
EE (%)	5.2
CF (%)	3.3
Calcium (%)	0.8
Phosphorus (%)	0.6
Sodium (%)	0.2

DE, Digestible energy; ME, Metabolizable energy; CP, Crude protein; EE, Ether extract; CF, Crude fibre.

## Data Availability

Not applicable.
